# 

**Published:** 1888-08

**Authors:** 


### TABLE OF CONTENTS.

### Original and Selected Articles.

Acute psoas abscess discharged through an opening into the ascending colon, by J. McF. Gaston, M. D, Atlanta, Georgia ............ 281
Baltimore Academy of Medicine—a case of uncontrollable epistaxis ............ 287
Note on the use of chloride of methyl, by Wm. M. Thallon, M.D. ............ 291
The curability of morphia addiction ............ 295

### Abstracts and Gleanings.

The treatment of tymphanites by puncture.... 297
Chloride of sodium in the sickness of pregnancy; treatment of heat fever or sunstroke. 298
Clinical Morphologies ............ 299
Agaracine ............ 300
Another triumph of laboratory research—tasteless preparations of sagrada ............ 301
Poisoning with cocaine ............ 303
Peppermint water in pruritus pudendi; a case of mammary cancer treated by inoculation with erysipelas ............ 304
Naptnol as an antiseptic medicament ............ 305
A cure of tuberculosis; experience in the use of cocaine ............ 306
Abdominal surgery; acid indigestion; paraldehyde in obstinate vomiting ............ 307
Kephir and its use as an infant food; intravascular injections in collapse; thymus vulgaris in the treatment of whooping-cough... 308
Treatment of penetrating gun-shot wounds of the cranium; the New York Colleges; impure antipyrin; strychnine in alcoholism... 309
Man's power of imagination; effects of atropia on the suckling child; veratrum viride; tonsillitis ............ 310

### Scientific Items.

Long distance telegraphy; a gigantic oak...... 311
Simple method of artificial respiration ............ 312

### Practical Notes and Formulæ.

A hypnotic for use in alcoholism; for vomiting in cholera infantum; to stop toothache; Cocaine cotton ............ 313
Antipyretics ............ 314

### Editorials and Miscellaneous.

Divorces in Connecticut; California; the American Association of Obstetric and Gynecologists; a new therapeutic agent ............ 315
Medical aristocracy ............ 316
Emulating perfection ............ 317
Books and pamphlets received ............ 318
Cascara sagrada; Medical College of Ohio; Southern Medical College; Tulane Medical University; to medical students ............ 319
Special notes ............ 320

